# Low back pain is associated with sleep disturbance: a 3-year longitudinal study after the Great East Japan Earthquake

**DOI:** 10.1186/s12891-022-06106-x

**Published:** 2022-12-27

**Authors:** Yutaka Yabe, Yoshihiro Hagiwara, Yumi Sugawara, Ichiro Tsuji

**Affiliations:** 1 Department of Orthopedic Surgery, Tohoku University School of Medicine, 1-1 Seiryo-machi, Aoba-ku, 980-8574 Sendai, Miyagi Japan; 2 Department of Orthopaedic Surgery, Tohoku University School of Medicine, 1-1 Seiryo-machi, Aoba-ku, 980-8574 Sendai, Miyagi Japan; 3 Division of Epidemiology, Department of Health informatics and Public Health, Graduate School of Public Health, Tohoku University, 2-1 Seiryo- machi, Aoba-ku, 980-8575 Sendai, Miyagi Japan; 4 Division of Epidemiology, Department of Health Informatics and Public Health, Tohoku University Graduate School of Public Health, 2-1 Seiryo-machi, Aoba-ku, 980-8575 Sendai, Miyagi Japan

**Keywords:** Low back pain, Sleep disturbance, Great East Japan Earthquake, Longitudinal study

## Abstract

**Background:**

Low back pain and sleep disturbance are common health problems worldwide which are also commonly observed among people after natural disasters. These symptoms are well known to coexist, and recent reports have indicated that sleep disturbance is a risk factor for low back pain. However, the influence of low back pain on sleep disturbance has rarely been assessed; therefore, this study aimed to clarify the association of low back pain with sleep disturbance, especially focusing on the frequency of low back pain, using 3-year cohort data after the Great East Japan Earthquake.

**Methods:**

This study used the data obtained from people living in the disaster-affected areas after the Great East Japan Earthquake (*n* = 2,097). Low back pain and sleep disturbance were assessed at 4, 5, 6, and 7 years after the disaster. The frequency of low back pain was defined as the number of low back pain episodes at and before the evaluation time point and categorized into five groups such as absence, 1, 2, 3, and 4 at the fourth time point and four groups such as absence, 1, 2, and 3 at the third time point. Multivariate logistic regression analyses were conducted to assess the association of low back pain with sleep disturbance.

**Results:**

Low back pain was significantly associated with sleep disturbance, and the association was stronger in participants with more frequent low back pain (adjusted odds ratios [95% confidence intervals],1.46 [1.10–1.95] in “1”; 2.02 [1.49–2.74] in “2”; 2.38 [1.67–3.40] in “3”; and 4.08 [2.74–6.06] in “4” in the frequency of low back pain) (*P* for trend < 0.001). Furthermore, antecedent low back pain was significantly associated with new-onset sleep disturbance, and the association was robust in more frequent low back pain (adjusted odds ratios [95% confidence intervals],1.60 [1.05–2.44] in “1”; 1.96 [1.20–3.21] in “2”; and 2.17 [1.14–4.14] in “3” in the frequency of low back pain) (*P* for trend = 0.007).

**Conclusion:**

Our study showed that low back pain is strongly associated with sleep disturbance. Attention should be paid to low back pain to prevent and treat sleep disturbance, especially focusing on chronicity of low back pain.

## Background

Sleep disturbance is a common health problem worldwide. [1, 2] Sleep disturbance has been reported to often coexist with pain, [3, 4] and the association between sleep disturbance and pain has garnered attention. Low back pain (LBP) is one of the most common musculoskeletal pains, and its association with sleep disturbance has also been reported. Previous cross-sectional studies have shown that patients with LBP commonly complain of sleep disturbance, and patients with sleep disturbance have severe LBP. [5, 6] When considering the causal relationship between sleep disturbance and LBP, some longitudinal studies have further shown their association. [7–14] Most of these studies have assessed the influence of sleep disturbance on LBP and have shown that sleep disturbance is a risk factor for LBP and a predictor of poor recovery from LBP. [7–9, 11–14] In contrast, a previous study showed that antecedent LBP caused sleep disturbance. [10] Therefore, association between sleep disturbance and LBP is considered to be bidirectional; however, the influence of LBP on sleep disturbance has been rarely assessed and is unclear.

Moreover, sleep disturbance and LBP are common health problems after natural disasters. [15, 16] The Great East Japan Earthquake (GEJE) hit the northeastern coastal areas of Japan on March 11, 2011. [17] After the GEJE, a high prevalence of LBP and sleep disturbance was reported, [18, 19] and sleep disturbance was associated with the continuation and new onset of LBP in a dose-dependent manner. [20, 21] However, the influence of LBP on sleep disturbance has not been investigated after natural disasters. Clarifying the association between LBP and sleep disturbance is crucial in developing prevention or treatment strategies for them. This study aimed to elucidate the association between LBP and sleep disturbance, especially focusing on the frequency of LBP and association of antecedent LBP with new-onset sleep disturbance using 3-year cohort data after the GEJE.

## Materials and methods

### Study design and participants

**Fig. 1:**
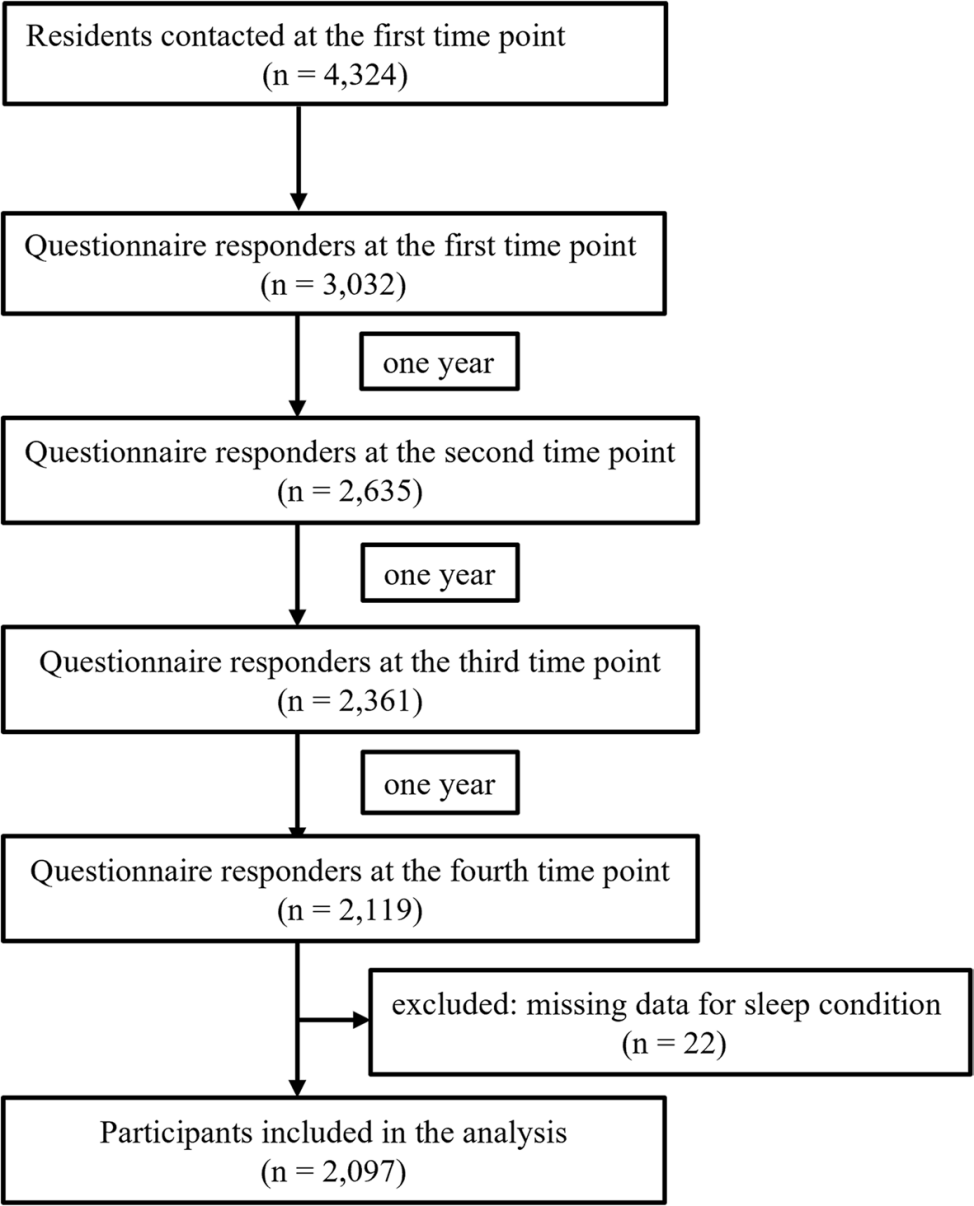
Flow chart of the study

The present study used one part of the data of a cohort study conducted among people living in disaster-affected areas after the GEJE, such as Ogatsu, Oshika, and Ajishima areas in Ishinomaki City and Wakabayashi ward in Sendai City, Japan. [15, 18, 22] This cohort aimed to assess the mental and physical health conditions of the people living in these areas and to support them. It has been started 3 months after the GEJE and has been continued annually. The initial population of the cohort included all people living in the three areas in Ishinomaki City and people living in the prefabricated houses in Wakabayashi ward in Sendai City. To assess the association between LBP and sleep disturbance, the present study used the 3-year longitudinal study data of people (18 years or over) at 4 (defined as the first time point), 5 (second time point), 6 (third time point), and 7 (fourth time point) years after the GEJE because follow-up rates in these periods were comparatively high. Individuals who had participated in the previous survey were recruited. Self-reported questionnaires and informed consent forms were mailed to the participants (*n* = 4,324). The number of questionnaire responders at the first time point was 3,032 (70.1%), and the response rates at the second, third, and fourth time points were 86.9% (2,635/3,032), 89.6% (2,361/2,635), and 89.8% (2,119/2,361), respectively. Individuals with missing data on sleep conditions at the third or fourth time point were excluded (*n* = 22) because sleep disturbance at these time points were used for the analyses, and 2,097 were included in the present study (Fig. 1).

### Outcome variable (sleep disturbance)

Participants’ sleep conditions were assessed using the Athens Insomnia Scale (AIS). The AIS consists of eight questions, with each question rated from 0 to 3 (0, no problem at all; 3, very serious problem). Sleep disturbance was defined as a score of ≥ 6/24 on the AIS. [23] We used the information on sleep conditions at the fourth time point to assess the association between LBP and sleep disturbance and sleep conditions at the third and fourth time points to assess the influence of antecedent LBP on the onset of sleep disturbance.

### Main predictor (LBP)

LBP was assessed using a self-report questionnaire at four time points. Participants were asked if they had LBP in the last few days; they were classified into “absence” or “presence” of LBP groups at each time point. The frequency of LBP at the third time point was defined as the number of “presence” of LBP at the first, second, and third time points and categorized into four groups: absence, 1, 2, and 3. Furthermore, the frequency of LBP at the fourth time point was defined as the number of “presence” of LBP at the first, second, third, and fourth time points and was categorized into five groups: absence, 1, 2, 3, and 4.

### Covariates

The following variables at the third or fourth time points were included in the analysis as covariates because they were considered potential confounding factors: sex, age, body mass index, living area, smoking and drinking habits, comorbid conditions, working status, walking time per day, living status, economic conditions, psychological conditions, and social network. Psychological conditions were assessed using the Kessler Psychological Distress Scale-6 (K-6), which consists of six mental health questions rated from 0 to 4. Psychological distress was defined as a score of ≥ 10/24 on the K-6. [24] Social network was assessed using the Lubben Social Network Scale-6 (LSNS-6), which consists of six social network questions rated from 0 to 5. Social isolation was defined as a score of < 12/30 on the LSNS-6. [25] These variables were categorized as shown in Table 1.

### Statistical analysis

The chi-square test was used to compare covariates due to the absence or presence of LBP at the fourth time point. The association between LBP and sleep disturbance was assessed using crude and multivariate logistic regression analyses, and odds ratios (ORs) and 95% confidence intervals (95% CIs) were calculated. The outcome of interest was sleep disturbance at the fourth time point. First, the main predictor was set as LBP and its frequency at the fourth time point to assess the association between LBP and sleep disturbance. The variables at the fourth time point were used as covariates in the analysis. Second, participants without sleep disturbance at the third time point were selected, and the main predictor was set as LBP and its frequency at the third time point to assess the influence of antecedent LBP on the onset of sleep disturbance. The variables at the third time point were used as covariates in the analysis. SPSS (version 24.0: IBM Corp., Armonk, NY) was used for the analyses, and a *P*-value of < 0.05 was considered significant.

## Results

The prevalence of LBP at each time point was 26.3% (551/2,097), 25.1% (526/2,097), 26.6% (558/1,097), and 26.8% (561/2,097) at the first, second, third, and fourth time points, respectively. The prevalence of sleep disturbance at the third and fourth time points was 34.0% (712/2,097) and 33.2% (697/2,097), respectively. The baseline characteristics of the participants at the fourth time point are listed in Table 1. Participants with LBP were more likely to be smokers and drinkers and have comorbid conditions such as hypertension and myocardial infarction, poor economic conditions, psychological distress, and social isolation. LBP was significantly associated with sleep disturbance at the fourth time point, and the adjusted OR (95% CI) was 2.21 (1.76–2.77), using the absence of LBP as a reference (*P* < 0.001) (Table 2). Furthermore, LBP frequency was significantly associated with sleep disturbance, and the adjusted ORs (95% CIs) were 1.46 (1.10–1.95) in “1,” 2.02 (1.49–2.74) in “2,” 2.38 (1.67–3.40) in “3,” and 4.08 (2.74–6.06) in “4” in the frequency of LBP at the fourth time point, using the absence of LBP as a reference (*P* for trend < 0.001) (Table 3).

Among the participants without sleep disturbance at the third time point, the prevalence of the onset of sleep disturbance at the fourth time point was 12.3% (171/1,385). LBP at the third time point was significantly associated with the onset of sleep disturbance at the fourth time point, and the adjusted OR (95% CI) was 1.83 (1.24–2.69), using the absence of LBP as a reference (P = 0.002) (Table 4). Additionally, the frequency of LBP at the third time point was significantly associated with the onset of sleep disturbance at the fourth time point, and the adjusted ORs (95% CIs) were 1.60 (1.05–2.44) in “1,” 1.96 (1.20–3.21) in “2,” and 2.17 (1.14–4.14) in “3” in the frequency of LBP at the third time point, using the absence of LBP as a reference (*P* for trend = 0.007) (Table 5).

**Table 1:** Baseline characteristics

		Low back pain			
		*n* (%)	absence	presence	*P* value
		2,097	1,536	561	
Sex	Male	931 (44.4)	677 (44.1)	254 (45.3)	0.624
	Female	1,166 (55.6)	859 (55.9)	307 (54.7)	
Age	< 65	826 (39.4)	613 (39.9)	213 (38.0)	0.421
	≥ 65	1,271 (60.6)	923 (60.1)	348 (62.0)	
Body mass index^a^	≥ 18.5, < 25	1,264 (60.3)	947 (61.7)	317 (56.5)	0.183
	< 18.5	38 (1.8)	27 (1.8)	11 (2.0)	
	≥ 25	720 (34.3)	511 (33.3)	209 (37.3)	
Living area	Ogatsu	879 (41.9)	642 (41.8)	237 (42.2)	0.698
	Oshika	750 (35.8)	544 (35.4)	206 (36.7)	
	Ajishima	141 (6.7)	109 (7.1)	32 (5.7)	
	Wakabayashi	327 (15.6)	241 (15.7)	86 (15.3)	
Smoking habits^a^	Non-smoker	1,687 (80.4)	1,256 (81.8)	431 (76.8)	0.015
	Smoker	334 (15.9)	233 (15.2)	101 (18.0)	
Drinking habits^a^	0 g of alcohol/day	1,317 (62.8)	988 (64.3)	329 (58.6)	0.031
	< 45.6 g of alcohol/day^b^	442 (21.1)	300 (19.5)	142 (25.3)	
	≥ 45.6 g of alcohol/day^b^	165 (7.9)	119 (7.7)	46 (8.2)	
Comorbid conditions	Hypertension	902 (43.0)	624 (40.6)	278 (49.6)	< 0.001
	Diabetes mellitus	224 (10.7)	155 (10.1)	69 (12.3)	0.147
	Myocardial infarction	140 (6.7)	81 (5.3)	59 (10.5)	< 0.001
	Cerebral stroke	35 (1.7)	26 (1.7)	9 (1.6)	0.889
Working status^a^	Unemployed	1,061 (50.6)	776 (50.5)	285 (50.8)	0.518
	Employed	982 (46.8)	724 (47.1)	258 (46.0)	
Walking time/day^a^	≥ 1 h	604 (28.8)	446 (29.0)	158 (28.2)	0.064
	30 min to < 1 h	763 (36.4)	579 (37.7)	184 (32.8)	
	< 30 m	701 (33.4)	489 (31.8)	212 (37.8)	
Living status^a^	Same house as before the GEJE	682 (32.5)	492 (32.0)	190 (33.9)	0.651
	Prefabricated house	86 (4.1)	60 (3.9)	26 (4.6)	
	New house	655 (31.2)	493 (32.1)	162 (28.9)	
	Others	647 (30.9)	472 (30.7)	175 (31.2)	
Economic condition^a^	Fair	1,040 (49.6)	812 (52.9)	228 (40.6)	< 0.001
	Poor	497 (23.7)	368 (24.0)	129 (23.0)	
	Poorer	327 (15.6)	200 (13.0)	127 (22.6)	
	Poorest	195 (9.3)	125 (8.1)	70 (12.5)	
Psychological distress^a^	Absence	1,801 (85.9)	1,357 (88.3)	444 (79.1)	< 0.001
	Presence	261 (12.4)	152 (9.9)	109 (19.4)	
Social isolation^a^	Absence	1,480 (70.6)	1,106 (72.0)	374 (66.7)	0.047
	Presence	616 (29.4)	429 (27.9)	187 (33.3)	

^a^Because each item has a limited number of respondents, the actual number is not necessarily in accordance with the total.

^b^22.8 g of alcohol amount to 1 go or traditional unit of sake (180ml), which also approximates to two glasses of wine (200ml), or beer (500ml) in terms of alcohol content. Categorical values are presented as numbers and percentages (%). GEJE indicates Great East Japan Earthquake.

**Table 2:** Association between low back pain and sleep disturbance at the fourth time point

	Low back pain			
	Total	Absence	Presence	*P* value
Participants	2,097	1,536	561	
Sleep disturbance, n (%)	697 (33.2)	425 (27.7)	272 (48.5)	
Crude OR (95% CI)		1 (Ref.)	2.46 (2.02-3.00)	< 0.001
Adjusted OR (95%CI)		1 (Ref.)	2.21 (1.76–2.77)	< 0.001

Adjusted for sex, age, body mass index, living area, smoking habits, drinking habits, comorbid conditions, working status, walking time, living status, subjective economic condition, psychological distress, and social isolation. OR indicates odds ratio; CI, confidence interval.

**Table 3:** Association between frequency of low back pain and sleep disturbance at the fourth time point

	Frequency of low back pain							
	Total	Absence	1	2	3	4	*P* for trend	
Participants	2,097	1,070	375	294	199	159		
Sleep disturbance, n (%)	697 (33.2)	256 (23.9)	125 (33.3)	120 (40.8)	97 (48.7)	99 (62.3)		
Crude OR (95%CI)		1 (Ref.)	1.59 (1.23–2.06)	2.19 (1.67–2.88)	3.02 (2.22–4.13)	5.25 (3.70–7.45)	< 0.001	
Adjusted OR (95%CI)		1 (Ref.)	1.46 (1.10–1.95)	2.02 (1.49–2.74)	2.38 (1.67–3.40)	4.08 (2.74–6.06)	< 0.001	

Adjusted for sex, age, body mass index, living area, smoking habits, drinking habits, comorbid conditions, working status, walking time, living status, subjective economic condition, psychological distress, and social isolation. OR indicates odds ratio; CI, confidence interval.

**Table 4:** Association between preceding low back pain and onset of sleep disturbance

	Low back pain at the third time point			
	Total	Absence	Presence	*P* value
Participants without sleep disturbance at the third time point	1,385	1,108	277	
Onset of sleep disturbance at the fourth time point, n (%)	171 (12.3)	120 (10.8)	51 (18.4)	
Crude OR (95% CI)		1 (Ref.)	1.86 (1.30–2.66)	< 0.001
Adjusted OR (95%CI)		1 (Ref.)	1.83 (1.24–2.69)	0.002

Adjusted for sex, age, body mass index, living area, smoking habits, drinking habits, comorbid conditions, working status, walking time, living status, subjective economic condition, psychological distress, and social isolation. OR indicates odds ratio; CI, confidence interval.

**Table 5:** Association between frequency of preceding low back pain and onset of sleep disturbance

	Frequency of low back pain at the third time point					
	Total	Absence	1	2	3	*P* for trend
Participants without sleep disturbance at the third time point	1,385	868	276	166	75	
Onset of sleep disturbance at the fourth time point, n (%)	171 (12.3)	86 (9.9)	41 (14.9)	29 (17.5)	15 (20.0)	
Crude OR (95%CI)		1 (Ref.)	1.59 (1.06–2.37)	1.93 (1.22–3.04)	2.27 (1.24–4.18)	0.003
Adjusted OR (95%CI)		1 (Ref.)	1.60 (1.05–2.44)	1.96 (1.20–3.21)	2.17 (1.14–4.14)	0.007

Adjusted for sex, age, body mass index, living area, smoking habits, drinking habits, comorbid conditions, working status, walking time, living status, subjective economic condition, psychological distress, and social isolation. OR indicates odds ratio; CI, confidence interval.

## Discussion

The present study revealed that LBP was associated with sleep disturbance, and this association was robust in participants with more frequent LBP episodes. Furthermore, antecedent LBP was associated with the onset of sleep disturbance, and the influence was stronger in participants with more frequent LBP episodes.

Sleep disturbance has been reported to coexist with LBP. [5, 6, 26, 27] Most of these studies have assessed sleep disturbance in patients with LBP. People with chronic LBP tend to have difficulty initiating sleep, reduced sleeping time, and lower sleep efficiency. [28] The rate of sleep disturbance among patients with chronic LBP was reported to be 50–60%. [6, 26] In the present study, 48.5% of the participants with LBP had sleep disturbance, even though LBP was not limited to chronic LBP; however, the rate was higher compared to 27.7% among the participants without LBP. Moreover, the association between LBP and sleep disturbances was significant after adjusting for potential confounding factors. Although several factors such as sex, age, and psychological and socioeconomic factors are associated with both LBP and sleep disturbance, [29–32] LBP is considered to be independently associated with sleep disturbance. In addition, previous studies have shown that LBP intensity correlates with the severity of sleep disturbance. [27, 33] These studies indicated that LBP and sleep disturbance are associated in a dose-dependent manner. Therefore, it is speculated that the frequency of LBP further affects the association between LBP and sleep disturbance. However, to the best of our knowledge, no study has assessed the association between LBP and sleep disturbance due to the frequency of LBP. The present study clearly showed that the association between LBP and sleep disturbance is stronger in patients with more frequent LBP. LBP is associated with sleep disturbance, and this association is considered robust among people with chronic LBP.

Regarding the causal relationship between LBP and sleep disturbance, some longitudinal studies have shown that antecedent sleep disturbance is associated with the onset of LBP among healthcare workers, [14] firefighters, [8] people after a natural disaster, [21] and the general population. [9] Additionally, other reports have shown that sleep disturbance is associated with poor recovery from pain in patients with LBP. [7, 12, 13] These reports indicate that sleep disturbance is a risk factor for LBP. However, only a few longitudinal studies have assessed the influence of LBP on sleep disturbances. Morelhão et al. reported that high LBP intensity in older patients was associated with poor sleep quality 6 months later. [10] The present study also showed that antecedent LBP was significantly associated with the onset of sleep disturbance 1 year later, even after adjustment for potential confounding factors. Although there have been only a few reports assessing the influence of pain on sleep disturbance, previous studies have shown that prior pain severity predicts subsequent sleep disturbance among patients with rheumatoid arthritis or orofacial pain. [34, 35] Furthermore, people with musculoskeletal pain, including LBP, would have a higher rate of sleep disturbance compared with those without the pain 1 year later. [19, 36] Regarding the influence of pain on sleep disturbance, it is hypothesized that pain prevents the initiation or continuation of sleep. [37] In addition, brain structure controlling nociception modulates sleep states, [38] and pain and sleep disturbance can occur due to a common neurobiological dysfunction. [37] A previous longitudinal study showed that preceding sleep disturbance was associated with onset of LBP, and the effect was stronger along with longer duration and increased frequency of sleep disturbance. [21] Conversely, the present study is the first to show that the influence of antecedent LBP on the onset of sleep disturbance is robust in participants with more frequent LBP. LBP leads to the onset of sleep disturbance in a dose-dependent manner, such as intensity [10] and frequency of LBP, as shown in this study. Sleep disturbance is considered a prospective symptom among people with LBP, and the onset of sleep disturbance can lead to poor recovery from LBP, [7, 13] and this interaction is assumed to be stronger dose-dependently. Understanding the mutual relationship between LBP and sleep disturbance is critical to prevent and treat these symptoms, especially focusing on chronicity.

This study has some limitations. First, LBP was assessed using a self-reported questionnaire, and pain was not quantified. The intensity of LBP is considered to affect sleep disturbance, which should be examined in future studies. Second, LBP and sleep disturbance were assessed at four time points over 3 years, and those for periods other than these time points were not clear, which may have affected the results. Finally, participants were people living in areas affected by the GEJE. Although 4 years have passed since the GEJE, the effects of the disaster may remain, and the generalizability of the results of the present study is not clarified.

In conclusion, LBP was associated with sleep disturbance among people living in places affected by the GEJE, and the association was robust in those with more frequent LBP. Furthermore, antecedent LBP was associated with the onset of sleep disturbance, and the effect was stronger in patients with more frequent LBP.

## Data Availability

All data generated or analyzed during this study are not publicly available due to ethical concerns, but are available from the corresponding author on reasonable request.

## Abbreviations

LBP: Low back pain
GEJE: Great East Japan Earthquake
OR: Odds ratio
95% CI: 95% confidence interval
