# Correction: Low back pain is associated with sleep disturbance: a 3-year longitudinal study after the Great East Japan Earthquake

**DOI:** 10.1186/s12891-023-06141-2

**Published:** 2023-01-10

**Authors:** Yutaka Yabe, Yoshihiro Hagiwara, Yumi Sugawara, Ichiro Tsuji

**Affiliations:** 1 Department of Orthopaedic Surgery, Tohoku University School of Medicine, 1-1 Seiryo-machi, Aoba-ku, Sendai, Miyagi 980-8574 Japan; 2 Division of Epidemiology, Department of Health informatics and Public Health, Tohoku University, Graduate School of Public Health, 2-1 Seiryo-machi, Aoba-ku, Sendai, Miyagi 980-8575 Japan

**Correction: BMC Musculoskelet Disord 23, 1132 (2022)**

10.1186/s12891-022-06106-x

Following publication of the original article [1], the authors identified an error in affiliations. Affiliation 1 is a duplicate of affiliation 2; and affiliation 3 is a duplicate of affiliation 4.

The affiliations have been updated above and the original article [1] has been corrected.
